# Mechanistic Fatigue Performance Evaluation of Stone Mastic Asphalt Mixtures: Effect of Asphalt Performance Grade and Elastic Recovery

**DOI:** 10.3390/polym16172414

**Published:** 2024-08-26

**Authors:** Jongsub Lee, Sungjin Lee, Yujoong Hwang, Ohsun Kwon, Gyumin Yeon

**Affiliations:** 1 Pavement Research Division, Korea Expressway Corporation Research Institute, Hwaseong 18489, Republic of Korea; jlee@ex.co.kr (J.L.); hyj@ex.co.kr (Y.H.); pooh2461@ex.co.kr (O.K.); 2 Joongbu Division North Gyeonggi Center, Korea Conformity Laboratories, Chuncheon 24341, Republic of Korea; bluelife@kcl.re.kr

**Keywords:** asphalt performance grade, elastic recovery, cyclic fatigue index parameter, fatigue cracking, asphalt mixture performance tester

## Abstract

This study evaluates the crack performance of stone mastic asphalt (SMA) mixtures according to the performance of a modified asphalt binder, evaluated based on the asphalt performance grade (PG) and the elastic recovery of multiple stress creep and recovery (MSCR) according to AASHTO M 320 and T 350. The cracking performance of the mixture was evaluated using the asphalt mixture performance tester (AMPT) according to AASHTO T 378 and T 400 through dynamic modulus and direct tension cyclic fatigue tests. Furthermore, the recently developed viscoelastic continuum damage (VECD) theory was utilized to evaluate the cyclic fatigue index parameter (apparent damage capacity, Sapp) and the permissible heavy vehicle class. For performance evaluation, six modified asphalt mixtures were prepared and tested using SMA aggregate gradation with a nominal maximum aggregate size (NMAS) of 10 mm. The MSCR test results revealed that, of the six asphalt mixtures, the rubber-based PG76-28 exhibited the least initial strain and the highest elastic recovery. The dynamic modulus test results demonstrated that using a rubber-based modifier increased the elastic modulus at high temperatures and decreased it at low temperatures, thereby enhancing resistance to plastic deformation in the summer and reducing low-temperature cracking in the winter. Finally, the correlation between the Sapp performance index and the elastic recovery of modified asphalt and the number of direct tension cyclic loads until failure of the mixture was evaluated as 0.87 and 0.76, respectively.

## 1. Introduction

Modified asphalt binder use is increasing, owing to its ability to extend the lifespan of asphalt concrete pavement. However, using modifiers has become necessary to enhance the performance of asphalt that has deteriorated through depressurization refining processes for the additional production of high-priced LPG, gasoline, diesel, and kerosene [1]. Modifiers improve the performance of asphalt binders by increasing their viscosity at high temperatures. This improvement enhances their resistance to plastic deformation while decreasing viscosity at low temperatures, thus improving cracking resistance. Furthermore, the modifier envelops the solid asphaltene component of the asphalt binder, thereby enhancing its elasticity. Thus, establishing asphalt performance evaluation and quality criteria is necessary to determine the appropriate type and amount of modifier to employ during the asphalt mixture design phase. In addition, quality control testing methods and standards are needed to quickly evaluate the performance of modified asphalt during asphalt plant production.

The Superpave mix design system was developed through the Strategic Highway Research Program (1994). From this system, the Asphalt Performance Grade Specification was introduced, which is based on the historical climate of a region [2,3]. However, it has been observed that the performance of modified asphalt binders with the same performance grade (PG) can vary significantly depending on the type and amount of modifier. Therefore, the multiple stress creep recovery (MSCR) testing method for evaluating the performance of modified asphalt was developed to address this limitation [4]. The U.S. Federal Highway Administration (FHWA) successfully validated the MSCR method using accelerated pavement testing and test roads. The correlation between the modified asphalt pavement and plastic strain in MSCR was evaluated to be significantly high at 82%. Furthermore, through the Mississippi test construction, a strong correlation of 75% was observed between MSCR testing and plastic strain [5]. Moreover, previous research showed that asphalt mixtures using modified asphalt with high elastic recovery exhibited greater fatigue crack resistance [6].

According to the study by Rieksts et al. (2018), the type and gradation of filler influence the rheological properties of asphalt mastics. This impact extends to key properties like elastic recovery and permanent deformation resistance, underscoring the importance of selecting appropriate filler materials in asphalt mix design [7].

The asphalt mixture performance tester (AMPT) was developed to evaluate the cracking resistance of asphalt mixtures under the National Cooperative Highway Research Program (NCHRP) Project 9–19, “Superpave Support and Performance Models Management”, and Project 9–29, “Simple Performance Tester for Superpave Mix Design” [8,9]. Recently, the FHWA utilized the test results of the AMPT to develop the cyclic fatigue index parameter (Sapp), which represents the effects of elastic modulus and toughness of asphalt mixtures based on the mechanical theory of the simplified viscoelastic continuum damage (S-VECD) model. The Sapp index is used to evaluate how changes in finer aggregate gradation, asphalt content, field compaction, and recycled aggregate amount affect the overall mix performance and its fatigue crack resistance. By assessing these factors, the Sapp index provides insights into optimizing mix design for improved pavement durability. Furthermore, the Sapp index provides a fatigue crack resistance rating based on traffic volume and congestion conditions [10].

This study aims to (1) evaluate the fatigue cracking performance of stone mastic asphalt (SMA) mixtures based on the PG and MSCR elastic recovery of modified asphalt, using the AMPT test and Sapp index and (2) determine the relationships among Sapp, the elastic recovery of modified asphalt, and the number of cycles to failure of the modified mixture.

## 2. Experimental Program and Analysis Methods

### 2.1. Material and Mix Design

**Figure 1:**
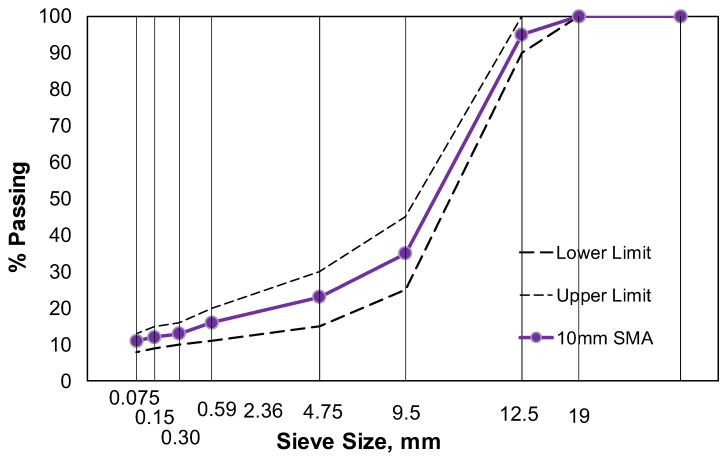
Aggregate gradation of 10 mm SMA.

This study used an SMA mixture with a nominal maximum aggregate size (NMAS) of 10 mm, commonly used in the surface layer of highway asphalt pavements, as mandated by the Korea Expressway Corporation guidelines [11]. The aggregate gradation shown in Figure 1 was used with a granite aggregate with the properties listed in Table 1. In Table 1, the criteria for modified asphalt mixtures are based on the specifications outlined in the technical document KCS 44 55 15: 2016 Aggregates [12]. Six types of asphalt binders, namely, PG64-22, 76-22A, 76-22B, 76-22C, 76-28, and 82-22, were utilized. In addition, the straight asphalt of type 1 was used, along with four types of rubber-based modifiers and a plastic-based modifier. In this study, PG76-28, PG82-22, and PG76-22 asphalt binders were modified using Styrene–Butadiene–Styrene (SBS) modifiers. The mass ratios of the modifiers were 4.5%, 4.25%, and 3.5% of the asphalt mass, respectively. All mixtures were produced in a laboratory setting to ensure precise control over the modification process. Cellulose fibers were added at a concentration of 0.5% to prevent the flow and bleeding of the SMA mixture.

The asphalt content was determined to be 6.9%, and it met the criteria for the voids in mineral aggregates (VMAs) and voids filled with asphalt (VFAs) in Table 2. The dynamic stability test results, which evaluated the resistance to plastic deformation, indicate that using PG64-22 asphalt without a modifier did not meet the quality criteria. Furthermore, PG76-22, PG76-28, and PG82-22 were evaluated to have better resistance to plastic deformation than PG76-22.

The PG test for straight and modified asphalt was conducted according to AASHTO M 320. The results of the PG tests for six types of asphalt binders using dynamic shear and bending beam rheometers are presented in Table 3.

Table 3 presents the performance grade (PG) test results for six types of asphalt binders. The G∗/sin(δ) values indicate the binders’ resistance to rutting at high temperatures, essential for maintaining stability under heavy traffic. The Pressure Aging Vessel (PAV) results show how the binders withstand long-term aging, providing insights into their durability. The stiffness and m-values at low temperatures reveal the binders’ flexibility and resistance to thermal cracking, critical for performance in cold climates. These metrics help determine the most suitable binders for various environmental and traffic conditions. In Table 3, the ‘↑’ symbol indicates that the value must be equal to or greater than the specified threshold, while the ‘↓’ symbol indicates that the value must be equal to or less than the specified threshold [3].

### 2.2. Testing Protocol and Analysis Methods

MSCR tests to measure elastic recovery were conducted to evaluate the performance of six types of asphalt, namely, PG 64-22, 76-22A, 76-22B, 76-22C, 76-28, and 82-22. Furthermore, dynamic modulus and direct tensile fatigue crack tests were conducted to evaluate the crack performance of six types of SMA mixtures. The test results of these mixtures were mechanically evaluated for their fatigue cracking performance using the cyclic fatigue index parameter (Sapp).

**Figure 2:**
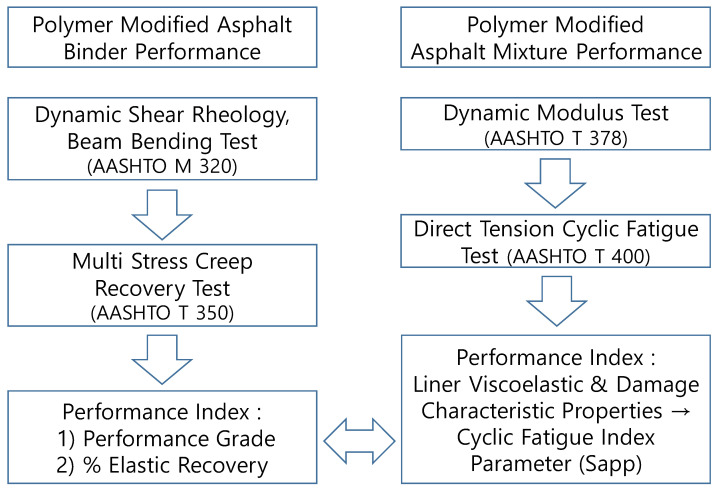
Structure of the experimental work.

The experimental and analytical procedures for evaluating the performance of modified asphalt and modified SMA mixtures in this study are presented in Figure 2.

### 2.3. Specimen Preparation

**Figure 3:**
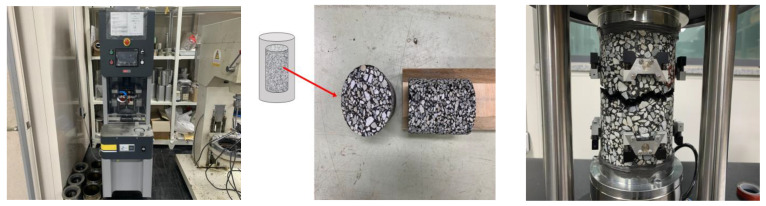
Gyratory compactor and specimen fabrication and middle failure of direct tension cyclic fatigue test.

The dynamic modulus and direct tension cyclic fatigue tests were conducted using a gyratory compactor to produce specimens with a diameter of 150 mm and a height of 180 mm. These specimens were then cored and cut to create test specimens with a diameter of 100 mm and heights of 150 mm and 130 mm, respectively, as shown in Figure 3. According to previous studies, when the height of fatigue crack specimens was reduced from the existing 150 mm to 130 mm, an increased occurrence rate of cracks was observed in the middle failure within the linear variable displacement transducer (LVDT) due to a consistent void distribution in the specimen [13,14].

### 2.4. Testing Protocol

The elastic recovery of the modified asphalt binder was tested using a dynamic shear rheometer according to AASHTO T 350. A test was conducted to stabilize the specimen at a temperature of 64 °C, where a creep shear stress of 0.1 kPa was applied for 1 s, followed by a 9 s rest period. This test was conducted 20 times to measure the elastic recovery. Subsequently, a test to measure the elastic recovery was conducted 10 times, where a creep stress of 3.2 kPa was applied for 1 s, followed by a 9 s rest period [4].

The dynamic modulus test of the mixture was conducted according to AASHTO T 378. LVDTs with a diameter of 70 mm were attached to cylindrical specimens with a diameter of 100 mm and a height of 150 mm. The specimens were subjected to loads within the 50–75 µε range through a trial-and-error process. The elastic modulus was measured at test temperatures of 5 °C, 20 °C, 40 °C, and 54.4 °C and at loading frequencies of 25, 10, 5, 1, 0.5, and 0.1 Hz to determine the master curve [15].

The fatigue crack test was conducted according to AASHTO T 400 using a direct tension cyclic fatigue test at 18 °C and a load frequency of 10 Hz. The test was performed on specimens with on-specimen LVDTs measuring strains ranging from 270 to 420 µε. The specimens were subjected to approximately 1000, 10,000, and 100,000 cycles until failure occurred. The point at which the phase angle, which represents the shift between stress and strain, reaches its peak, was determined as the location of the fracture during the test. Before the fatigue crack test, a fingerprint test of the dynamic modulus was conducted on all specimens at 18 °C and 10 Hz to measure the variability between the specimens [16].

### 2.5. Analysis Method

**Figure 4:**
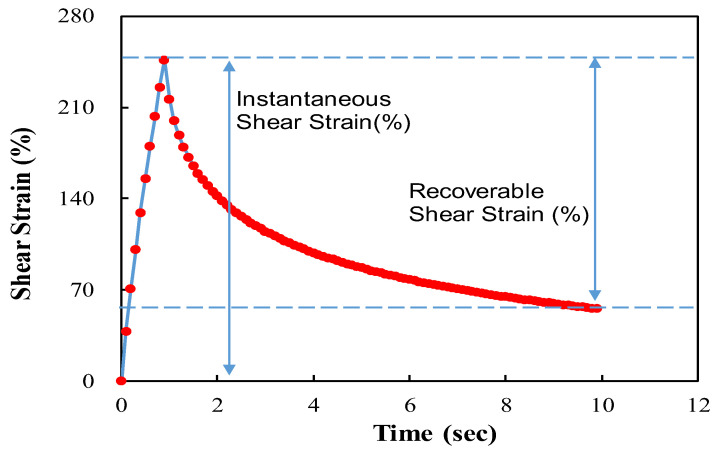
Elastic recovery by MSCR test.

The method for measuring the elastic recovery of modified asphalt is the same as shown in Figure 4. The recoverable shear strain is evaluated as the ratio of the instantaneous shear strain caused by a 1 s load to the shear strain during a 9 s rest period. The final elastic recovery of modified asphalt is calculated as the average value of 10 test cycles.

(1) $$\%\;\mathrm{Elastic}\;\mathrm{Recovery}=\frac{recoverable\;shear\;strain}{instantaneous\;shear\;strain}$$

A mechanical analysis method using the viscoelastic continuum damage model was employed to evaluate the fatigue cracking performance of the modified asphalt mixture. In continuum damage mechanics, the lifespan of a material is represented as the amount by which it decreases compared to its initial stiffness. This mechanical model enables the prediction of the initiation point of material cracks at a given temperature and load by determining the damage characteristic curve of the material and the failure criterion of D^R^. This study utilized the principles of linear elastic–viscoelastic correspondence, time–temperature superposition, Schapery’s work potential theory, and the failure criterion of average reduction in material integrity up to failure to apply the evaluation of crack performance based on continuum damage theory to asphalt mixtures [17,18,19,20,21]. This study used the Sapp, developed by the FHWA based on the continuum damage theory, to evaluate the crack performance of modified SMA mixtures. The equation for determining Sapp is as follows:

(2) $$\mathrm{Sapp}=1000^{\frac{\alpha }{2}-1}\times \frac{{a_{T}}^{\frac{1}{\alpha +1}}\;\times \;{\left(\frac{D^{R}}{C11}\right)}^{\frac{1}{C12}}}{{\left|E^{*}\right|}^{\frac{\alpha }{4}}}$$

where

α = damage evolution rate, maximum slope of relaxation modulus;

$a_{T}$ = time-temperature shift factor at 10 Hz, 18 °C;

$\left|E^{*}\right|$ = dynamic modulus of mixture at 10 Hz, 18 °C in kPa;

C11, C12 = coefficients of damage characteristic curve model;

$D^{R}$ = failure criterion, average reduction in material integrity up to failure.

Table 4 presents the parameters applied for each performance grade (PG) to calculate Sapp. The linear viscoelastic properties, damage evolution rate, and time–temperature shift factor values obtained from the dynamic modulus test, as well as the elastic modulus value at a load rate of 10 Hz and 18 °C, and the fitting coefficients of the power function model for the damage characteristic curve obtained from the direct tension cyclic fatigue test, were used to calculate the Sapp using the average decrease in material integrity as the failure criterion. Here, the fatigue cracking test temperature was 18 °C, which is the average value of the asphalt PG at a high temperature of 64 °C and a low temperature of −22 °C minus 3 °C based on the local climate.

In addition, the FHWA proposed the fatigue cracking resistance grade based on traffic volume and vehicle speed using Sapp, as shown in Table 5. The criteria within Table 5 were determined by comparing the indoor test results of AMPT for a total of 105 mixtures, including general, recycled, medium-temperature, and modified asphalt mixtures, with the performance of asphalt pavements in field use, test roads, and accelerated pavement testing.

## 3. Discussion of Results

This section describes the results of elastic recovery for straight asphalt and five types of modified asphalt based on the modifier content and PG. Additionally, the text presents the results of dynamic modulus and direct tension cyclic fatigue tests conducted on SMA mixtures comprising six types of asphalt. It also includes an analysis of the damage characteristic curve, failure criterion, and Sapp using an established mechanical model. Finally, the relationships between the Sapp, the elastic recovery of asphalt binder MSCR, and the number of cycles to failure in direct tension cyclic fatigue tests of SMA mixtures were evaluated.

### 3.1. Asphalt Elastic Recovery

**Figure 5:**
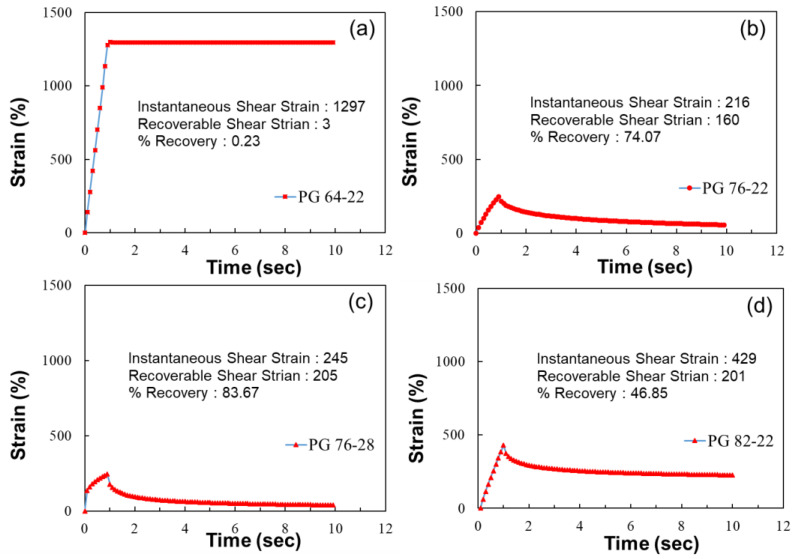
Instantaneous and recoverable shear strains depending on the asphalt PG: (**a**) PG64-22, (**b**) PG76-22, (**c**) PG 76-28 and (**d**) PG82-22.

The test results for MSCR according to the PG rating are shown in Figure 5. The straight asphalt binder without any modifiers, as shown in Figure 5a, was evaluated to have no recovery in deformation during the rest period after a 1 s load application. 

The elastic recovery rates of asphalt containing modifiers were found to be 83.67% for PG 76-28, 74.07% for PG 76-22, and 46.85% for PG 82-22. Additionally, the instantaneous shear strain was reduced by an average of 77% compared to straight asphalt when modifiers were included. Using modifiers reduced the initial deformation of the pavement under traffic loads and increased its resilience to initial changes.

**Figure 6:**
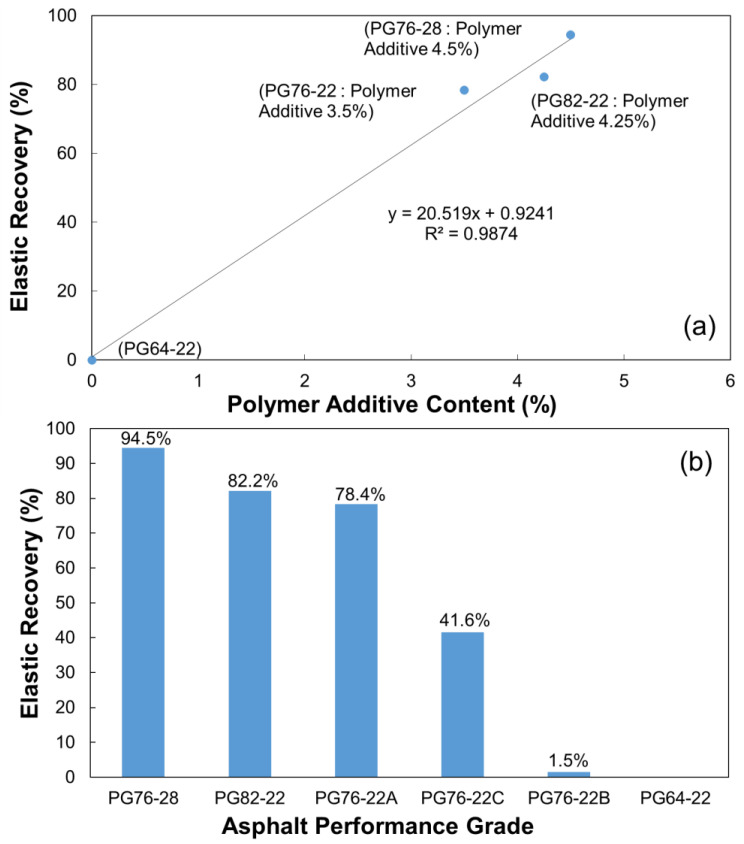
Elastic recovery depending on the (**a**) PA content and (**b**) asphalt PG.

Figure 6a shows an increase in elastic recovery as the concentration of the modifier increases. It was observed that the elastic recovery increased by approximately 21% for each 1% increase in polymer additive content. This demonstrates that the quality control evaluation of modified asphalt using rubber modifiers can be performed through elastic recovery. 

In addition to the four types of modified asphalt from the SBS series used in Figure 5, the elastic recoveries of two other types of modified asphalt, namely, the PG76-22 rating, applied with different types of rubber and plastic modifiers, were also evaluated. As shown in Figure 6b, a significant difference was observed in the elastic recovery of MSCR depending on the type of modifier (A, B, and C), even for the same PG 76-22 rating.

### 3.2. Dynamic Modulus Test of the SMA Mixtures

**Figure 7:**
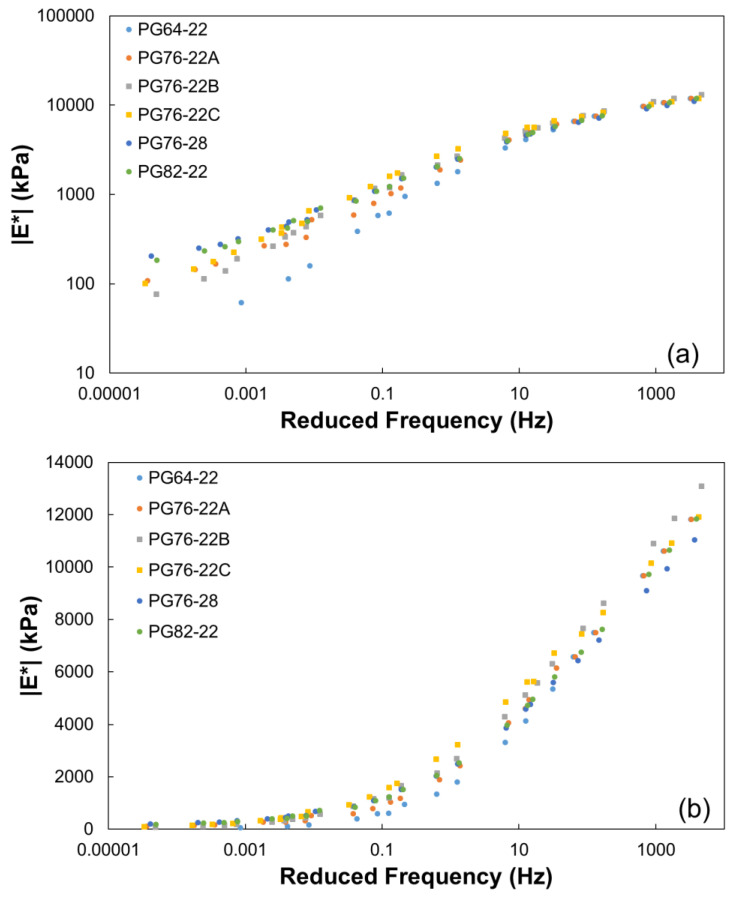
Dynamic modulus master curve: (**a**) log-log and (**b**) semi-log scales.

The dynamic modulus test results of the SMA mixtures showed that the LVDT detached from the specimen during testing at 54.4 °C, making it impossible to measure the dynamic modulus of the asphalt of PG64-22 without the modifier. This demonstrates that in SMA mixtures with a high asphalt content of 6.9%, the asphalt flows at high temperatures if a modifier is not used. As shown in Figure 7, PG64-22 was evaluated to have the lowest elastic modulus at high and ambient temperatures compared to the asphalt with modifiers.

Furthermore, PG76-28, which contains the largest amount of rubber modifier, had the largest elastic modulus at high temperatures and the smallest at low temperatures. This suggests that PG76-28 can reduce the risk of low-temperature cracking as it has the smallest pavement displacement due to its load in the summer and is soft in the winter.

### 3.3. Direct Tension Cyclic Fatigure Test of SMA Mixtures

**Figure 8:**
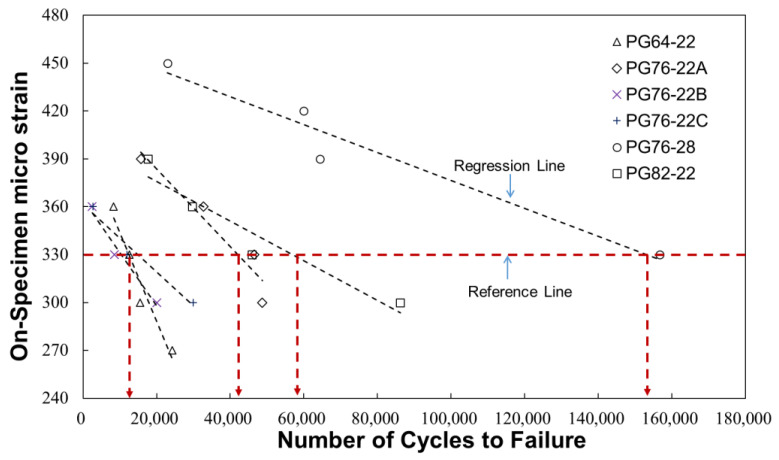
Direct tension cyclic fatigue test results of SMA mixtures.

The direct tension cyclic fatigue test results for six SMA mixtures, namely, PG 64-22, 76-22A, 76-22B, 76-22C, 76-28, and 82-22, are presented in Figure 8. The SMA mixture of PG 76–28 has been evaluated as having the best fatigue crack resistance among all strains. The on-specimen LVDTs were used to detect fracture failure in the specimens, which occurred under direct tension cyclic fatigue testing conditions of approximately 330 με, within approximately 160,000 cyclic loads. Furthermore, previous studies analyzing the pavement structure revealed that the subgrade behavior of typical asphalt pavement is evaluated within the 200–350 µε range [22].

### 3.4. Damage Characteristic Curve and Failure Criteria of SMA Mixtures

**Figure 9:**
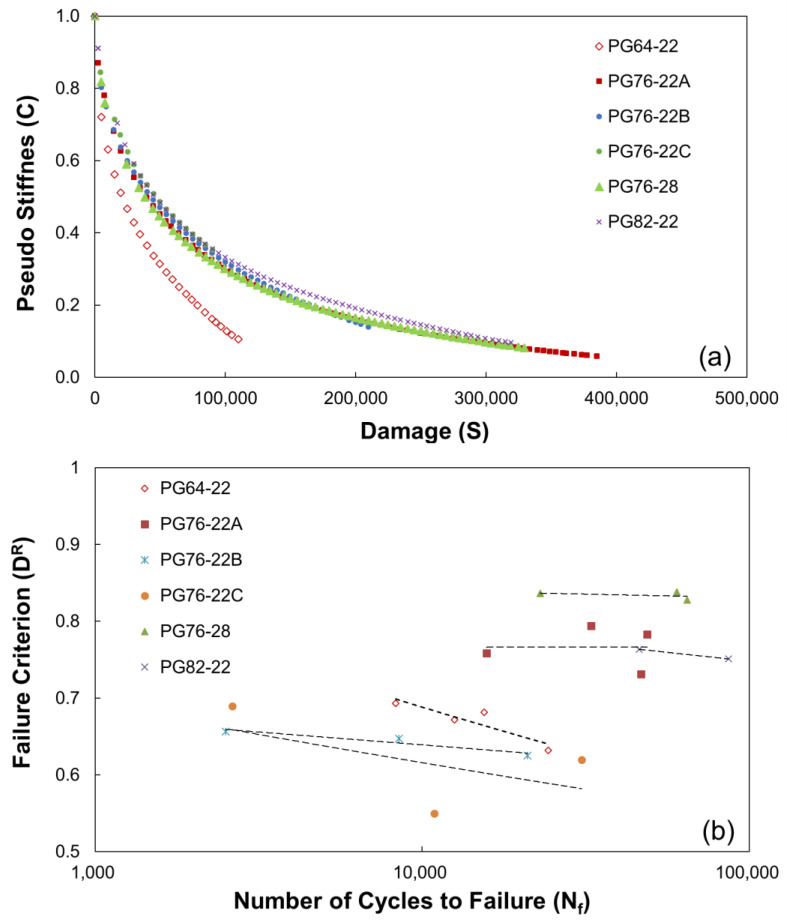
(**a**) Damage characteristic curve and (**b**) average reduction in material integrity up to failure.

The damage characteristic curve was evaluated for SMA mixtures through direct tension cyclic fatigue tests and elastic modulus tests, as shown in Figure 9a. This mechanical characteristic demonstrates how the initial pseudo-stiffness (C) decreases as damage (S) accumulates.

The analysis results demonstrate that PG64-22, which does not contain a modifier, exhibits a significant decrease in mixture stiffness, owing to the accumulation of damage caused by cyclic loading. The remaining modified SMA mixtures were evaluated to have similar damage–characteristic curve shapes. However, the accumulated damage at the time of breakage varies depending on the damage curve length for each mixture. Figure 9b illustrates the correlation between the number of cycles to failure for each mixture and the destruction criterion D^R^. The fatigue crack resistances of the mixtures, as indicated by the decreasing rate of integrity, showed the following descending order: PG76-28, PG76-22A, PG82-22, PG64-22, PG76-22B, and PG76-22C.

### 3.5. Cyclic Fatigue Index Parameter (Sapp) of SMA Mixtures

**Figure 10:**
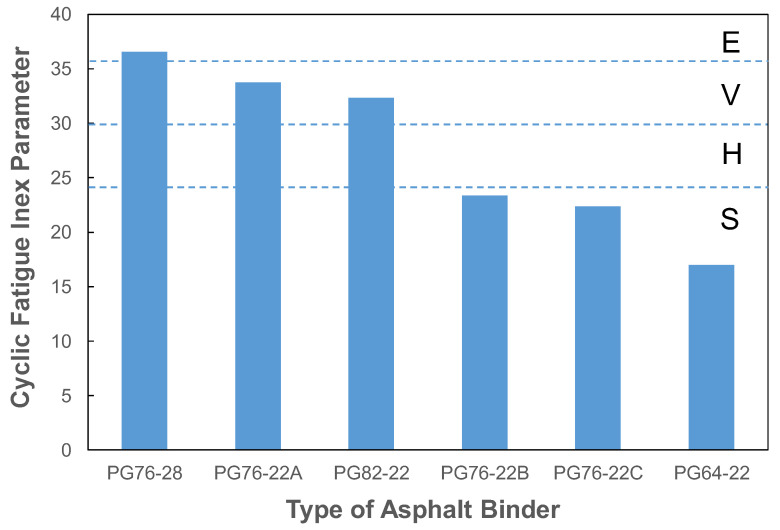
Cyclic fatigue index parameter results of SMA mixtures.

Sapp is a mechanistic crack resistance index that considers the structural behavior of the pavement using a master curve analyzed through elastic modulus testing, as well as the stiffness and toughness of materials analyzed through direct tension cyclic fatigue testing. Figure 10 evaluates the Sapp according to the PG of the 10 mm SMA mixture. PG 76-28 was rated as the highest with an E-grade of 36.5, while PG 76-22A and PG 82-22 were rated as V-grade with 33.7 and 32.3, respectively. The remaining PGs, PG 76-22B, PG 76-22C, and PG 64-22, were evaluated as S-grade with the lowest scores of 23.4, 22.3, and 17.0, respectively.

### 3.6. Relationship between Asphalt Elastic Recovery, Nf, and Sapp

**Figure 11:**
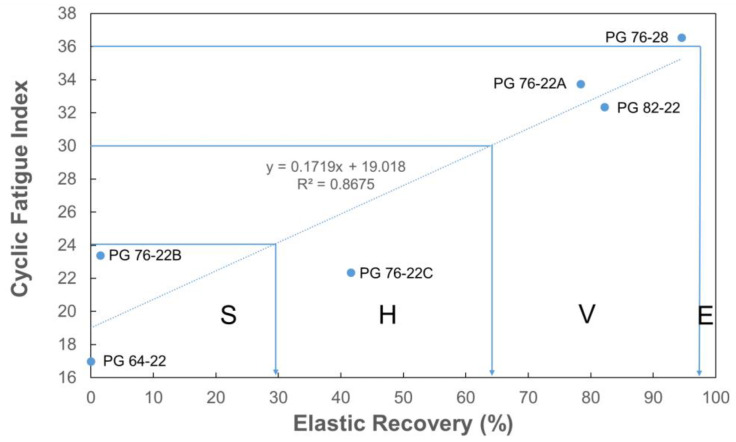
Relationship between the cyclic fatigue index and asphalt elastic recovery.

**Figure 12:**
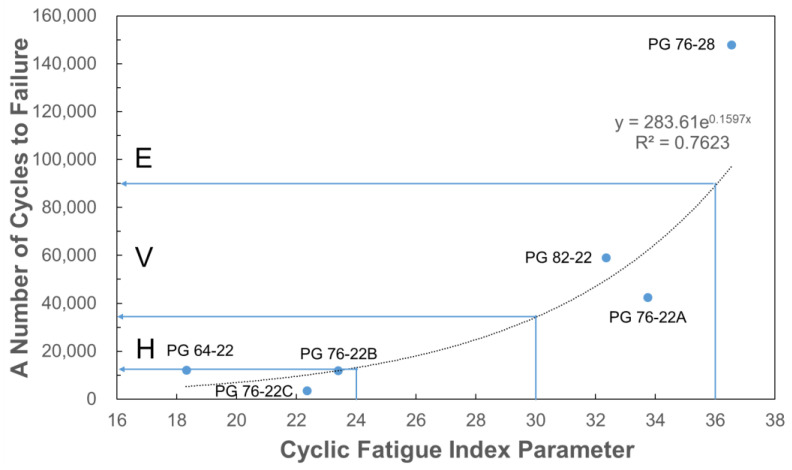
Relationship between the cyclic fatigue index and Nf of mixtures.

Finally, the relationships between the Sapp of the mixture, the elastic recovery of asphalt, and the number of direct tension cycles to failure of the mixture were determined, as shown in Figure 11 and Figure 12.

The elastic recoveries for each grade, as evaluated in comparison to the H, V, and E ratings proposed by the FHWA, were approximately 30%, 65%, and 98% or higher, respectively. The correlation between Sapp and elastic recovery was found to have an R-squared value of 0.87. This means that the elastic recovery test of MSCR can be used as a quality control index for modified asphalt during production.

The number of direct tension cycles to failure of the mixture was evaluated to be approximately 13,000, 34,000, and 89,000 cycles or higher, respectively, corresponding to heavy vehicle traffic Sapp rating criteria of H, V, and E. Furthermore, the correlation between Sapp and the number of cycles to failure was observed to have an R-squared value of 0.76. This indicates that the number of cycles to failure of a mixture can be used to evaluate the fatigue crack resistance of the mixture during the mixture design phase via additional tests.

## 4. Conclusions

The findings of this study are as follows:

(1) When a modifier was added to straight asphalt, the initial shear strain decreased, and the recoverable shear strain increased. The sensitivity analysis showed that elastic recovery increased by approximately 21% for each 1% increase in polymer additive content.
(2) The rubber-based PG 76-28 asphalt was evaluated to have the lowest initial strain (245) and the highest elastic recovery (83.67%) among the six types of asphalt.
(3) Even with the modified asphalt of the same PG 76-22 rating, a significant difference was observed in the elastic recovery depending on the performance of the modifier.
(4) Determining the appropriate modifier amount for elastic recovery testing was possible when rubber-based modifiers were used. Thus, this can be utilized as a quality control test for modified asphalt.
(5) When a rubber-based modifier was used in straight asphalt, the elastic modulus increased at high temperatures and decreased at low temperatures, thereby enhancing resistance to plastic deformation in the summer and reducing low-temperature cracking in the winter.
(6) In the Sapp evaluation according to the PG of the 10 mm SMA mixture, PG 76-28 was rated as the highest with an E-grade of 36.5, while PG 76-22A and PG 82-22 were rated as V-grade with 33.7 and 32.3, respectively. The remaining PGs, PG 76-22B, PG 76-22C, and PG 64-22, were evaluated as S-grade with the lowest scores of 23.4, 22.3, and 17.0, respectively.
(7) The correlations between the Sapp and the modified asphalt elastic recovery, and between the Sapp and the number of direct tension cycles to failure of the mixture, were evaluated to be 0.87 and 0.76, respectively.
(8) When applying the permissible heavy vehicle traffic and congestion rating criteria of Sapp, the elastic recoveries of modified asphalt in the H, V, and E sections of a 10 mm SMA mixture were approximately 30%, 65%, and 98% respectively. The number of direct tension cycles to failure of the mixture was evaluated to be at least 13,000, 34,000, and 89,000 cycles or higher, respectively, for 330 µε using an on-specimen LVDT.

Our research highlights that the MSCR elastic recovery test can be used as a quality control index for modified asphalt during production. Additionally, the number of fatigue crack failures in mixtures can be used as a test standard for evaluating fatigue crack resistance during the mix design phase. These findings suggest a practical and straightforward evaluation method that can streamline quality control and ensure the durability of modified asphalt mixtures under various conditions.

## Figures and Tables

**Table 1:** Aggregate properties.

Item	True Density (g/cm^3^)	Density in Saturated Surface Dry Condition (g/cm^3^)	Density in Absolute Dry Condition (g/cm^3^)	Absorption Rate (%)	Abrasion Loss (%)
Criterion	-	-	Above 2.5	Below 3.0	Below 30
Coarse Aggregate	2.706	2.659	2.631	1.061	21.5
Fine Aggregate	2.614	2.600	2.591	0.328	-

**Table 2:** Mix design results of 10 mm SMA mixtures depending on PGs.

List of Mix Design	Criteria	Results		
		PG64-22	PG76-22/28	PG82-22
Asphalt Binder Content (%)	above 6.6	6.9		
Design Air Void (%)	2–4	3.2	3.0	2.8
Design VMA (%)	above 18	18.9	18.8/18.7	18.0
Design VFA (%)	above 75	83.2	84.0	88.3
Drain Down (%)	below 0.3	0.12	0.14	0.18
Dynamic Stability (cycles/mm)	above 2500	352	6721/7417	12,378

**Table 3:** PG test results of six types of asphalt binders.

Aged (Criterion)	Unit	PG62-22	PG76-22A	PG76-22B	PG76-22C	PG76-28	PG82-22
Orig. G*/sinδ (1.0 ↑)	kPa	1.35 (64 °C)	1.57 (76 °C)	1.29 (76 °C)	2.26 (76 °C)	1.91 (76 °C)	2.45 (82 °C)
		0.64 (70 °C)	1.00 (82 °C)	0.78 (82 °C)	1.22 (82 °C)	1.26 (82 °C)	1.08 (88 °C)
RTFO G*/sinδ (2.2 ↑)	kPa	2.43 (64 °C)	2.33 (76 °C)	2.28 (76 °C)	2.77 (76 °C)	2.32 (76 °C)	4.32 (82 °C)
		1.14 (70 °C)	1.42 (82 °C)	1.36 (82 °C)	1.57 (82 °C)	1.50 (82 °C)	2.66 (88 °C)
PAV G* × sinδ (5000 ↓)	kPa	2815 (25 °C)	1266 (31 °C)	1926 (31 °C)	2748 (31 °C)	542 (28 °C)	1998 (34 °C)
Stiffness (300 ↓)	MPa	145 (−12 °C)	175 (−12 °C)	224 (−12 °C)	174 (−12 °C)	40 (−18 °C)	152 (−12 °C)
m-value (0.3 ↑)	-	0.33 (−12 °C)	0.31 (−12 °C)	0.30 (−12 °C)	0.32 (−12 °C)	0.32 (−18 °C)	0.33 (−12 °C)

**Table 4:** Sapp parameters by performance grade.

Sapp Parameters	PG62-22	PG76-22A	PG76-22B	PG76-22C	PG76-28	PG82-22
α	3.03	3.71	3.54	3.75	4.17	4.22
$a_{T}$	2.408	2.444	2.546	2.551	2.424	2.499
$\left|E^{*}\right|$	5,773,000	6,739,000	6,163,000	6,330,000	4,344,000	5,788,000
C11	0.0147	0.0143	0.0068	0.0057	0.0154	0.0071
C12	0.352	0.332	0.399	0.416	0.336	0.387
$D^{R}$	0.67	0.77	0.66	0.69	0.83	0.76

**Table 5:** Recommended threshold value for the fatigue index parameter by FHWA [10].

Traffic (Million ESALs)	Saap Limits	Grade	Designation
<10	Saap > 8	Standard	S
10≤, ≤30	Saap > 24	Heavy	H
>30	Saap > 30	Very Heavy	V
>30, slow traffic	Saap > 36	Extremely Heavy	E

## Data Availability

The original contributions presented in the study are included in the article, further inquiries can be directed to the corresponding author/s.

## References

[1] Federal Highway Administration (2024). Asphalt Production and Oil Refining. https://pavementinteractive.org/reference-desk/materials/asphalt/asphalt-production-and-oil-refining/.

[2] (2006). Volumetric Requirements for Superpave Mix Design.

[3] (2023). Standard Specification for Performance-Graded Asphalt Binder.

[4] (2023). Standard Method of Test for Multiple Stress Creep Recovery (MSCR) Using a Dynamic Shear Rheometer (DSR).

[5] (2011). The Multiple Stress Creep Recovery Procedure.

[6] Yan C., Yuan L., Yu X., Ji S. (2022). Characterizing the Fatigue Resistance of Multiple Modified Asphalts Using Time Sweep Test, LAS Test and Elastic Recovery Test. Constr. Build. Mater..

[7] Rieksts K., Pettinari M., Haritonovs V. (2018). The influence of filler type and gradation on the rheological performance of mastics. Road Mater. Pavement Des..

[8] (2011). Simple Performance Tester for Superpave Mix Design.

[9] (2006). Superpave Support and Performance Models Management.

[10] (2019). Cyclic Fatigue Index Parameter (Sapp) for Asphalt Performance Engineered Mixture Design.

[11] Korea Expressway Corporation (2013). Improvement Plan for Asphalt Overlay on Concrete Pavements. https://www.codil.or.kr/viewDtlCostSave.do?gubun=costsave&pMetaCode=EDGCODA00933.

[12] (2016). Aggregates.

[13] (2022). Standard Practice for Preparation of Cylindrical Performance Test Specimens Using the Superpave Gyratory Compactor (SGC).

[14] Lee J.S., Norouzi A., Kim Y.R. (2017). Determining Specimen Geometry of Cylindrical Specimens for Direct Tension Fatigue Testing of Asphalt Concrete. J. Test. Eval..

[15] (2017). Standard Method of Test for Determining Dynamic Modulus and Flow Number for Asphalt Mixtures Using the Asphalt Mixture Performance Tester (AMPT).

[16] (2023). Standard Method of Test for Determining the Damage Characteristic Curve of Asphalt Mixtures from Direct Tension Cyclic Fatigue Tests.

[17] Kim Y.R., Lee Y., Lee H.J. (1995). Correspondence Principle for Characterization of Asphalt Concrete. J. Mater. Civ. Eng..

[18] Chehab G., Kim Y.R., Schapery R.A., Witczack M., Bonaquist R. (2002). Time-Temperature Superposition Principle for Asphalt Concrete Mixtures with Growing Damage in Tension State. J. Assoc. Asphalt. Paving Technol..

[19] Underwood B.S., Kim Y.R., Guddati M.N. (2010). Improved Calculation Method of Damage Parameter in Viscoelastic Continuum Damage Model. Int. J. Pavement Eng..

[20] Kim Y.R., Guddati M. (2012). Hot Mix Asphalt Performance-related Specifications Based on Viscoelastoplastic Continuum Damage (VEPCD) Models.

[21] Wang Y.D., Underwood S. (2020). Development of a Fatigue Index Parameter, *S_app_*, for Asphalt Mixtures using Viscoelastic Continuum Damage Theory. Int. J. Pavement Eng..

[22] Lee J., Lee J., Kwon S., Kim Y.R. (2013). Use of Cyclic Direct Tension Tests and Digital Imaging Analysis to Evaluate Moisture Susceptibility of Warm Mix Asphalt Concrete. Transp. Res. Rec. J. Transp. Res. Board.

